# *Baylisascaris procyonis* Parasites in Raccoons, Costa Rica, 2014

**DOI:** 10.3201/eid2208.151627

**Published:** 2016-08

**Authors:** Mario Baldi, Gilbert Alvarado, Steve Smith, Mario Santoro, Natalie Bolaños, Carlos Jiménez, Sabine E. Hutter, Chris Walzer

**Affiliations:** University of Veterinary Medicine, Vienna, Austria (M. Baldi, S. Smith, S.E. Hutter, C. Walzer);; Universidad Nacional, Heredia, Costa Rica (M. Baldi, N. Bolaños, C. Jiménez);; Universidad de Costa Rica, San Pedro, Costa Rica (G. Alvarado);; Istituto Zooprofilattico Sperimentale del Mezzogiorno, Naples, Italy (M. Santoro)

**Keywords:** Baylisascaris procyonis, parasites, larva migrans, Costa Rica, eco-epidemiology, public health, raccoons, Procyon lotor

**To the Editor:** *Baylisascaris procyonis* (Ascaridoidea: Ascarididae) parasites are facultatively heteroxenous nematodes that are widely distributed in the United States and Canada, where prevalence rates reach 70%–90%. They colonize the small intestine of their final host, the northern raccoon (*Procyon lotor*), whose feces can contain up to 25 × 10^3^ eggs/g. Under ideal environmental conditions (100% humidity and 24°C), eggs become infective in soil (*1**,**2*). When ingested by other mammalian hosts, third-stage larvae can produce pathologic changes called larva migrans, which can lead to chronic neurologic disorders and even death (*1*,*3*). *B. procyonis* parasite infection of humans occurs by the fecal–oral route (ingestion of eggs in contaminated food) (*1*). Small children are particularly vulnerable through accidental geophagia. Public health concerns arise where raccoon and human populations overlap.

As elsewhere, raccoons in Costa Rica have expanded their range into human-dominated areas, becoming common in the Greater Metropolitan Area, an ≈2,000-km^2^ portion of the Central Valley, home to 2.6 million persons. During the past decade, the government wildlife agency (Ministerio de Ambiente y Energía [MINAE]) reported a steep increase in raccoon-related complaints (*4*).

We examined raccoons for which a nuisance complaint was received by MINAE at 8 locations inside the Greater Metropolitan Area and report the southernmost range extension of *B. procyonis* parasites (previously not detected at latitudes below 31° N; Costa Rica [8°–11° N] is substantially farther south [*2*]). *B. procyonis* parasites in kinkajous (*Potos flavus*) have been reported, but that parasite was subsequently determined to be *B. potosis* (*5**,**6*).

For 10 months in 2014, raccoons were trapped in wooded areas and residential gardens by using baited traps (Havahart, Lititz, PA, USA) over 315 trap-nights. Fecal samples were collected from the animals and from communal latrines near the trapping sites, and the Sheather flotation technique was used to detect eggs in the feces (*1*). During raccoon necropsies, any adult roundworms (including *B. procyonis*) found in the gastrointestinal tract were fixed in 70% and 100% ethanol for morphologic and molecular identification, respectively.

Parasites were examined by light microscopy. Those identified as *B. procyonis* were counted and sexed. Voucher specimens of *B. procyonis* were deposited in the Natural History Museum, London, UK (accession no. NHMUK 2015.2.23 1–2). Nematodes were assigned to the genus *Baylisascaris* on the basis of genus-specific features. Species-specific features of *B. procyonis* (shape of lip denticles, male pericloacal rough areas, and male tail shape [*7*]) were used to distinguish *B. procyonis* from *B. columnaris* (*6**,**7*). Eggs were identified according to size and shell thickness. The shell has a characteristic soft granular surface (*3*). Mean size of the oval eggs was 57.0 μm (range 59.34–55.48) by 70.3 μm (range 51.5–72.1) (*1**–**3*). 

We used DNA extracted from *B. procyonis* parasites to amplify the mitochondrial cytochrome c oxidase 2 gene, ribosomal ITS1–5.8S-ITS2, and ribosomal 28S genes by using the primers and protocol described by Franssen et al. (*8*). We found 100% identity between the sequences from *B. procyonis* parasites from Costa Rica and those from North America (GenBank accession nos. AF179908 [cytochrome c oxidase 2 region], JQ403615 [ITS1–5.8S-ITS2 region], and KC434770 [28S region]).

We found *B. procyonis* parasites in 10 of 20 captured raccoons (Table), from which 137 adult worms (78 females, 59 males) were recovered. Infection intensity was 1–60 parasites/raccoon (mean 12.5). Average specimen length was 11.6 cm (range 8.1–20 cm). *B. procyonis* infection was found in raccoons at all 8 locations.

**Table:** Age and sex of raccoons sampled for roundworm testing, Costa Rica, 2014

Raccoons	No. (%) raccoons	
	Sampled	Positive for *Baylisascaris procyonis* parasites
Age		
Juvenile	4 (20)	3 (30)
Adult	16 (80)	7 (70)
Sex		
M	6 (30)	2 (20)
F	14 (70)	8 (80)
Total	20	10

Our sampling locations included 2 playgrounds and 1 school yard. A previous study found high prevalence of *Toxocara* spp. nematode eggs in dog feces from the same geographic region (*9*). Because egg identification can be difficult and that study was based exclusively on morphologic description without molecular confirmation or electron microscopy, it is possible that some *B. procyonis* eggs were misidentified as *Toxocara* spp. Both *Toxocara* spp. and *B. procyonis* parasites can cause larva migrans, the latter being more aggressive. In the Greater Metropolitan Area and Costa Rica in general, free-ranging dogs are common, including at playgrounds and school yards, sites also vulnerable to nocturnal visits by raccoons. Dogs can have patent *B. procyonis* parasite infections and can play a role in transmission of the parasite from raccoons to humans.

In Costa Rica, cases of larva migrans have been reported. The Unidad de Investigación y Análisis, Registros y Estadísticas de Salud at the National Children’s Hospital, San José, Costa Rica, reported 135 cases of larva migrans ocularis and 21 cases of visceral larva migrans caused by nonspecifically identified ascarids during 2005–2014 (unpub. data). However, these diagnoses were based on IgG serologic testing results (Martinez J., National Children’s Hospital; pers. comm., 2015), which do not identify ascarid species. Western blot testing would improve accuracy (*10*).

The eco-epidemiology of *B. procyonis* parasites in tropical settings is relevant to public health because it might play a yet-unrecognized role in larva migrans pathology, which can be severe. Increased contact between raccoons and humans also warrants further investigation to improve understanding and minimize zoonotic risk.
